# Reinventing the echocardiography workflow: from manual quantification to artificial intelligence–driven comprehensive interpretation

**DOI:** 10.1186/s44348-026-00087-4

**Published:** 2026-08-26

**Authors:** Ran Heo, Seung-Ah Lee, Hyuk-Jae Chang

**Affiliations:** 1 Division of Cardiology, Department of Internal Medicine, Hanyang University Medical Center, Hanyang University College of Medicine, Seoul, Republic of Korea; 2 CONNECT-AI Research Center, Yonsei University College of Medicine, Seoul, Republic of Korea; 3 Ontact Health Inc, Seoul, Republic of Korea; 4 Division of Cardiology, Department of Internal Medicine, Severance Hospital, Yonsei University College of Medicine, Seoul, Republic of Korea

**Keywords:** Artificial intelligence, Echocardiography, Workflow, Global Longitudinal Strain, Point-of-care testing

## Abstract

Echocardiography remains the cornerstone of cardiovascular imaging. However, traditional workflows including manual acquisition, sequential measurement, and expert interpretation face challenges from increased clinical demand, workforce shortage, and the physical burden of repetitive scanning. Artificial intelligence (AI) has begun to address these issues, transitioning from proof-of-concept to prospective clinical evaluations. Recent evidence suggests that AI integration reduces examination time and automates measurements, enabling more comprehensive data collection while mitigating sonographer fatigue and improving image quality. The sonographer's role is accordingly evolving from conventional measurement to active verification. AI applications in echocardiography now extend beyond ejection fraction to integrated assessments of myocardial texture and Doppler hemodynamics. New model architectures incorporate both structural and functional evaluations, reflecting clinical reasoning of the expert. These methods are being applied to valvular heart disease, cardiomyopathy, and pericardial disorders. Clinical implementation of AI in echocardiography requires more than high accuracy. Current evidence is limited by reliance on single-center studies, inconsistent performance across platforms, and the potential for automation bias in high-volume settings. This review evaluates current evidence, identifies existing gaps, and outlines the requirements for responsible clinical implementation of AI.

## Background

Echocardiography is the most widely used cardiovascular imaging modality, valued for its real-time imaging capability, portability, and safety profile. A standard transthoracic echocardiogram (TTE) encompasses image acquisition by a trained sonographer, manual measurement of a wide range of structural and functional parameters, and comprehensive interpretation by a cardiologist. This workflow faces growing pressure from increasing referral volumes, workforce constraints, and the physical demands of repetitive scanning on sonographers [1].

The measurement process represents a major workflow bottleneck, requiring the generation of dozens of individual parameters depending on disease complexity. This manual, time-intensive task is subject to interobserver variability, leading to diagnostic inconsistency and potentially impacting clinical management.

Prior work had documented the influence of artificial intelligence (AI) across all stages of echocardiography—from acquisition guidance to automated measurement—while characterizing full workflow automation as a future goal [2].

In this narrative review, relevant studies were identified through targeted literature searches with an emphasis on prospective, randomized, and externally validated studies published between 2023 and 2026. A summary of main studies is listed in Table 1.

**Table 1:** Study-level summary of AI applications in echocardiography

Study	Study design	Sample size	Validation	AI task	Key finding	Main limitation
Workflow efficiency and measurement standardization						
Hollitt et al. [1] (2025)	Prospective, single-center crossover	35 Volunteers, 70 exams	Deployment^a^	View recognition + automated measurement	Keystrokes, 503 → 231; scan time, 18 min → 13.4 min	Single-center; healthy volunteers; vendor-affiliated author
Sakamoto et al. [3] (2026)	Randomized crossover	585 Exams, 4 sonographers	Deployment^a^	Automated measurement	Time 13.0 min vs. 14.3 min; parameters ↑ 3.4-fold	Not blinded to AI; screening-only population
He et al. [5] (2023)	Blinded RCT, noninferiority	3,495 Studies, 3,035 patients	External	Automated LVEF	Substantial change 16.8% (AI) vs. 27.2% (sonographer), P < 0.001	Single trial site; some authors are inventors
Tromp et al. [6] (2022)	Prospective noninferiority validation	602 Studies, 600 people	Internal	23-Parameter automated measurement	IEC < 0 all 23 parameters; analysis time 1 min	Funded by Us2.ai; multiple author COIs
Sahashi et al. [7] (2025)	Model development	Internal (derivation, 155,215; temporal split validation, 2,103); external, 1,158	Internal + external	18-Parameter open-source automation	Coverage probability: internal, 0.796; external, 0.839	Validation limited to two centers; additional out-of-distribution datasets lack ground truth
Szasz et al. [35] (2026)	Retrospective cross-platform comparison	116 Subjects	None^b^	Comparison of commercial AI platforms	AI-AI differences significant 6 of 10 parameters; AI variability ≤ inter-reader	Several authors employed by one evaluated vendor
Prognostic value of automated strain						
Merin et al. [13] (2026)	Retrospective, single center	731 Patients	None^b^	Automated strain for mortality prediction	AI vs. human mortality prediction similar (AUC, 0.67 vs. 0.66); AI + strain model superior (AUC, 0.73; P = 0.048)	Single center; heterogeneous cohort; 18% excluded
Multitask AI						
Holste et al. [14] (2025)	Model development	Internal, 32,265; external, ≈26,000 (4 datasets)	Internal + external	18 Classifications + 21 parameters, single model	Median AUC, 0.91; median normalized MAE, 0.13	2D/color Doppler only; no segmentation step
Valvular heart disease						
Sadeghpour et al. [15] (2025)	Retrospective multicenter model development	438 Patients	External	Automated MR severity grading	Accuracy 0.80 (severity); 0.97 (significant vs. not)	Predominantly secondary MR; industry ties
Vrudhula et al. [17] (2025)	Retrospective single-center model development	Internal (train, 47,312; test, 2,462); external, 5,549	Internal + external	Two-stage TR severity classification	AUC 0.928–0.980 (≥ moderate/severe TR)	Labels not adjudicated; single view only
Lin et al. [18] (2026)	Retrospective model development	MR cohort, n = 2,584; TR cohort, n = 2,502	Internal + external	End-to-end MR/TR severity pipeline	Internal AUC ≥ 0.980 (≥ moderate MR and TR); external 0.934–0.941(≥ moderate MR or TR); PPV drop for severe disease (0.50–0.54)	External severity distribution differed, degrading PPV for severe disease
Long et al. [19] (2025)	Retrospective multicenter model development	Internal, 61,659; external, 10,001	Internal + external	Multiview AR/MR/TR severity classification	AUC > 0.95 (≥ moderate, all valves); MR progression HR: internal, 4.1; external, 2.1	Color Doppler only; both sites same vendor; industry ties
Park et al. [20] (2025)	Multicenter model development	Internal, 8,427; external, 2,468 (2 datasets)	Internal + external	DL index for AS continuum + automated quantitation	AUC, 0.91–0.99 (any AS) to 0.97–0.99 (severe AS)	All-Korean data; several authors hold equity/patents
Cardiomyopathy and myocardial texture						
Ioannou et al. [21] (2026)	Multicenter model development	Internal, 4,371; external, 1,405 (2 sites)	Internal + external	Cardiac amyloidosis classification	Accuracy 87.5%–88.4% externally (AUC, 0.92–0.93)	≈13% Indeterminate output; industry ties
Moon et al. [24] (2025)	Multicenter model development	Internal, 867; external, 619	Internal + external	LVH etiology multiclass classification (HCM vs. CA vs. HHD vs. normal)	External AUC: HCM, 0.96; CA, 0.89; HHD, 0.86	All-Korean cohort; excludes rare etiologies; industry ties
Pericardial disease						
Chao et al. [25] (2024)	Single center, retrospective	Internal, 381; external 23	Internal + external	Binary CP vs. CA classification	Internal AUC, 0.97; external AUC, 0.84	CA as sole RCM surrogate; small external cases
Jeong et al. [26] (2026)	Multi-institution two-stage DL	Internal, 2,253; external, 274	Internal + external	Effusion grade + thickening/adhesion + hemodynamic significance	External AUC: 0.95–0.98 (effusion grade), 0.85 (thickening/adhesion), 0.76 (hemodynamic significance)	Modest hemodynamic-significance sensitivity; all-Korean
LLM-based reporting						
Syryca et al. [27] (2025)	Proof-of-concept, single center	21 Cases	None^b^	LLM-based report generation	Mean score 6.86 of 8; 85.7% fully acceptable	Small scale; text-only, no imaging input
Nonspecialist use, POCUS, and access						
Narang et al. [29] (2021)	Prospective multicenter	240 Patients	Deployment^a^	AI-guided acquisition by novices	Diagnostic-quality scans 98.8% (LV/effusion), 92.5% (RV)	Small number of operators (n = 8), no untrained-novice control group
Frydman et al. [30] (2026)	Prospective	281 Patients	Deployment^a^	AI-assisted bedside echo by nonspecialists	Change in care 32% (AI) vs. 20% (control); aOR, 2.16	Single center; not blinded to AI use
Campbell et al. [31] (2025)	Prospective multicenter	867 Patients	Deployment^a^	Fully automated LVEF quantification	Diagnostic accuracy 0.93 (AUC, 0.96) for LVEF ≤ 40%	LVEF reportable in only 61% of handheld scans
Papadopoulou et al. [32] (2024)	Prospective single center	115 Patients	Deployment^a^	Acquisition guidance + automated LVEF (nonexperts)	Correlation with reference EF (r): 0.90 (cardiologist) vs. 0.79–0.84 (staff); LVEF < 50% sensitivity/specificity 95%/94% (cardiologist) vs. 86%–95%/87%–93% (staff)	Nested case–control design; selected population

AI, artificial intelligence; AR, aortic regurgitation; AS, aortic stenosis; AUC, area under the curve; aOR, adjusted odds ratio; CA, cardiac amyloidosis; COI, conflict of interest; CP, constrictive pericarditis; DL, deep learning; EF, ejection fraction; HCM, hypertrophic cardiomyopathy; HHD, hypertensive heart disease; HR, hazard ratio; IEC, individual equivalence coefficient; LLM, large language model; LV, left ventricular; LVEF, left ventricular ejection fraction; LVH, left ventricular hypertrophy; MAE, mean absolute error; MR, mitral regurgitation; POCUS, point-of-care ultrasound; PPV, positive predictive value; RCM, restrictive cardiomyopathy; RCT, randomized controlled trial; RV, right ventricular; TR, tricuspid regurgitation

^a^The prospective clinical application of a previously developed/validated AI tool, without a train/test split. ^b^No train/test split and no external reference-standard validation (comparative or outcome-association study)

To avoid ambiguity across studies with different levels of automation, terminology in this review is used to reflect the level of autonomy of each application. “AI-assisted” refers to systems that support clinician-performed tasks, such as view guidance or measurement suggestion. “AI-integrated” refers to workflows in which AI is embedded across one or more steps of acquisition, measurement, interpretation, or reporting. “Fully automated” refers to task-specific outputs generated without manual execution, although clinical review may still be required. “Human-in-the-loop” refers to workflows in which clinicians explicitly verify, modify, or approve AI-generated outputs before they are incorporated into the final clinical report.

**Fig. 1:**
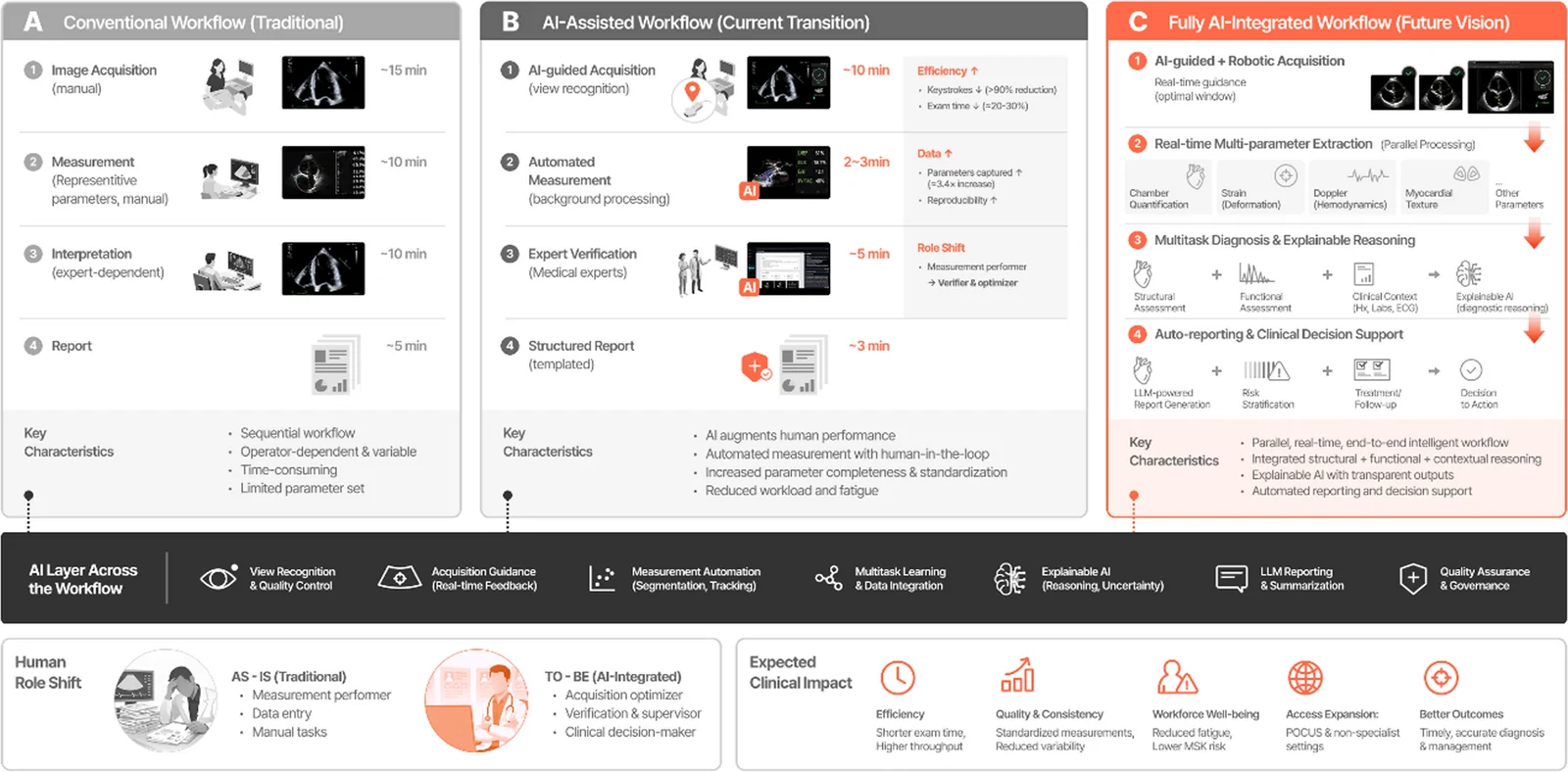
Overview of artificial intelligence (AI) integration across the echocardiography workflow. Conventional echocardiography follows a sequential workflow consisting of manual image acquisition, operator-dependent measurement, and expert interpretation. The integration of AI introduces automation across all stages, including real-time acquisition guidance, automated measurement, and structured reporting. This transition shifts the workflow from a sequential, operator-driven process to a more integrated and partially parallel system, while redefining the sonographer’s role toward active verification. Emerging AI-driven systems further extend this paradigm by enabling real-time multiparameter analysis, explainable diagnostic reasoning, and automated report generation, establishing a foundation for a fully integrated and intelligent echocardiographic workflow. LLM, large language model; POCUS, point-of-care ultrasound; MSK, musculoskeletal

**Fig. 2:**
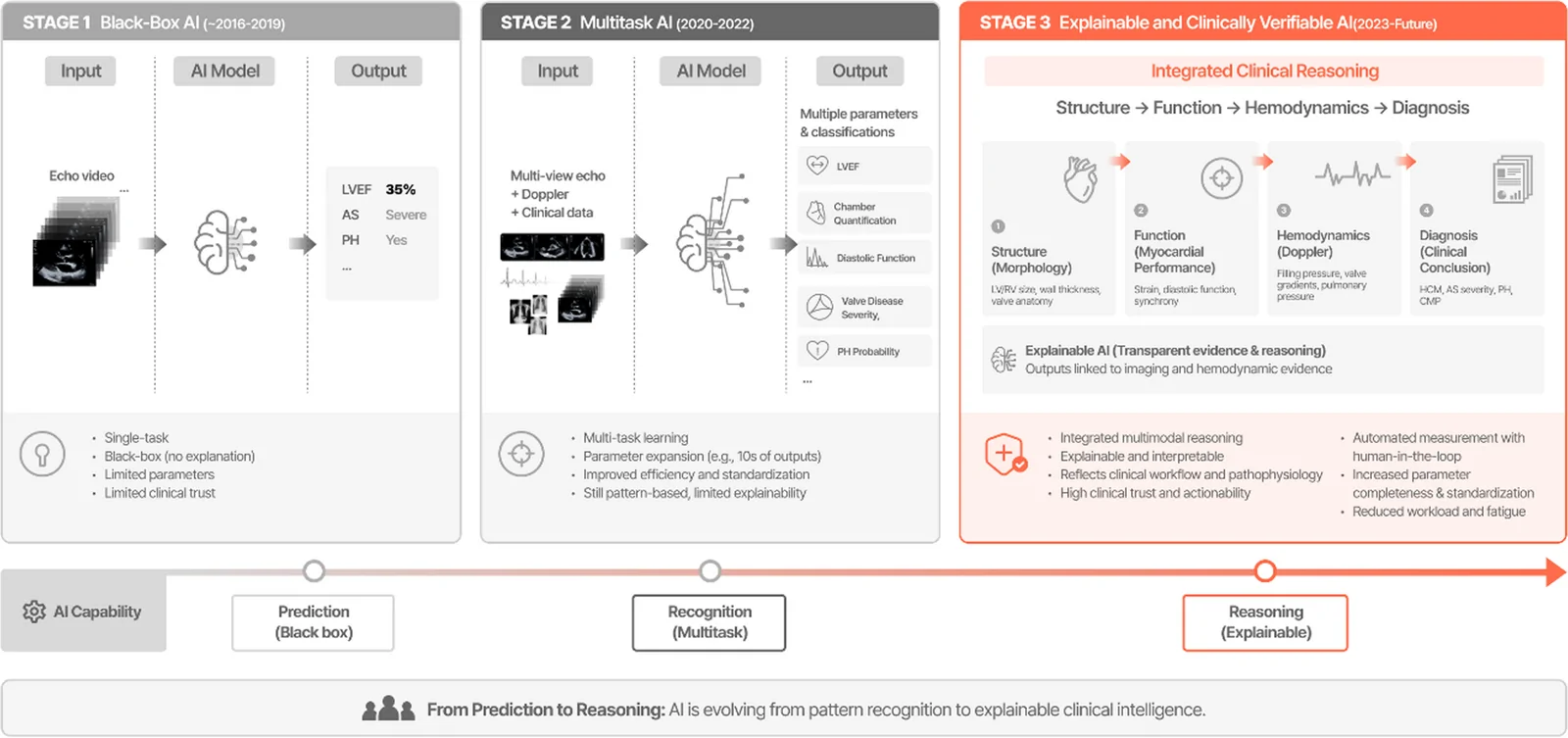
Evolution of artificial intelligence (AI) in echocardiography: from black-box prediction to explainable clinical reasoning. Early AI systems in echocardiography operated as black-box, single-task models that generated diagnostic predictions directly from imaging data with limited interpretability. Subsequent multitask models expanded capability by extracting multiple parameters and classifications from shared representations. More recent approaches extend beyond pattern recognition by integrating structural, functional, and hemodynamic information into explainable frameworks that reflect clinical reasoning processes. This evolution represents a shift from isolated prediction toward transparent, evidence-based diagnostic reasoning

This review examines the current evidence critically, assessing the achievement, limitation, and future goals for AI to deliver its potential within the clinical echocardiography laboratory. Across this evidence base, AI in echocardiography is moving from unexplained, black-box pattern recognition toward systems that formalize and externalize expert clinical reasoning. To frame this transition, the integration of AI across the echocardiography workflow is illustrated in Fig. 1, and the conceptual evolution it reflects—from black-box prediction, to multitask measurement, to explainable clinical reasoning—is summarized in Fig. 2, which provides the organizing framework for this review.

## Workflow efficiency and diagnostic standardization

AI's contribution to workflow efficiency and measurement standardization is now well established across multiple prospective studies, providing a mature foundation for the more diagnostically complex applications discussed in the next section.

### Physical burden and scanning efficiency

The repetitive nature of echocardiographic scanning—sustained probe positioning, frequent console interaction, and high keystroke volume—is a recognized driver of musculoskeletal disorders among sonographers. Hollitt et al. [1] showed that an AI-assisted protocol incorporating view recognition and automation-assisted measurement reduced console keystrokes from 503 to 231 and active scanning time from 18 to 13.4 min, without compromising image quality. Consolidating view recognition, cardiac phase selection, and caliper placement into a single automated step has been reported to reduce keystrokes for standard B-mode left ventricle (LV) measurements even further, illustrating the ergonomic gains AI-integrated protocols may achieve as they mature.

### Workflow efficiency

A randomized crossover trial in Japan compared AI-assisted versus conventional workflow across 38 study days and 585 screening examinations, with cardiologists finalizing all reports blinded to arm assignment [3]. AI assistance reduced examination time modestly (13.0 ± 3.5 min vs. 14.3 ± 4.2 min, *P* < 0.001) while the number of parameters measured per examination expanded 3.4-fold (*P* < 0.001), increasing daily throughput (16.7 ± 2.5 studies per day vs. 14.1 ± 2.5 studies per day, *P* = 0.003) and image quality (*P* < 0.001), with lower sonographer fatigue scores (4.1 ± 1.1 vs. 4.7 ± 0.6, *P* = 0.039) and 90% clinical concordance with expert-endorsed values. A separate fully automated system reduced analysis time from 490 to 94 s [4].

These findings suggest that AI shifts the expert role from active measurement to active verification—a change in cognitive demand whose governance implications (including the risk of automation bias) are addressed later in this review. A structured comparison of conventional and AI-integrated workflows is provided in Table 2.

**Table 2:** Comparison of conventional and AI-integrated echocardiography workflows

Domain	Conventional workflow	AI-integrated workflow
Acquisition	Manual, operator-dependent	AI-guided and quality-assisted acquisition
Measurement scope	Selective measurement of representative parameters	Comprehensive extraction of all available parameters
Workflow structure	Sequential processing	Partially parallelized processing
Data completeness	Incomplete and variable	Systematic and comprehensive
Variability	High interobserver and intraobserver variability	Reduced variability through standardization
Expert role	Direct performance of measurement and interpretation	Verification, supervision, and synthesis of AI-generated outputs
Cognitive demand	Procedural and repetitive	Supervisory and integrative
Time efficiency	Time-intensive	Reduced acquisition and processing time
Throughput	Limited	Increased
Advanced metrics (e.g., strain)	Not routinely acquired	Routinely incorporated by default

AI, artificial intelligence

### Measurement accuracy

#### LV systolic function

A blinded, randomized noninferiority trial found that LV ejection fraction (LVEF) changed substantially (> 5%) between initial and final cardiologist assessment in only 16.8% of AI-assessed studies, versus 27.2% for sonographer-assessed studies; cardiologists could not distinguish AI-generated from sonographer-generated assessments, and the AI-guided workflow saved time for both roles [5]. A separate pilot study of 40 TTE sets, including cases with poor image quality, found AI-derived LVEF met the noninferiority criterion against the cardiologist-adjudicated reference (intraclass correlation coefficient, 0.90) [4].

#### Formal multiparameter validation

In a formal validation of 602 studies, deep learning measurement of 23 echocardiographic parameters disagreed less with any individual human reader than the three human readers disagreed among themselves, meeting pre-specified noninferiority criteria for every parameter—evidence that automated measurement can standardize results beyond what operator-dependent measurement achieves [6].

#### Open-source comprehensive automation

Addressing generalizability, Sahashi et al. [7] developed an open-source deep learning model covering 18 anatomic and Doppler parameters, trained at Cedars-Sinai Medical Center and externally validated at Stanford Healthcare, with consistent performance across age, sex, atrial fibrillation status, obesity, and machine vendor. Open-source frameworks such as this provide a transparent benchmark for independent replication, particularly as commercial platforms increasingly rely on proprietary algorithms with limited interpretability.

## Advanced diagnostic capabilities

**Fig. 3:**
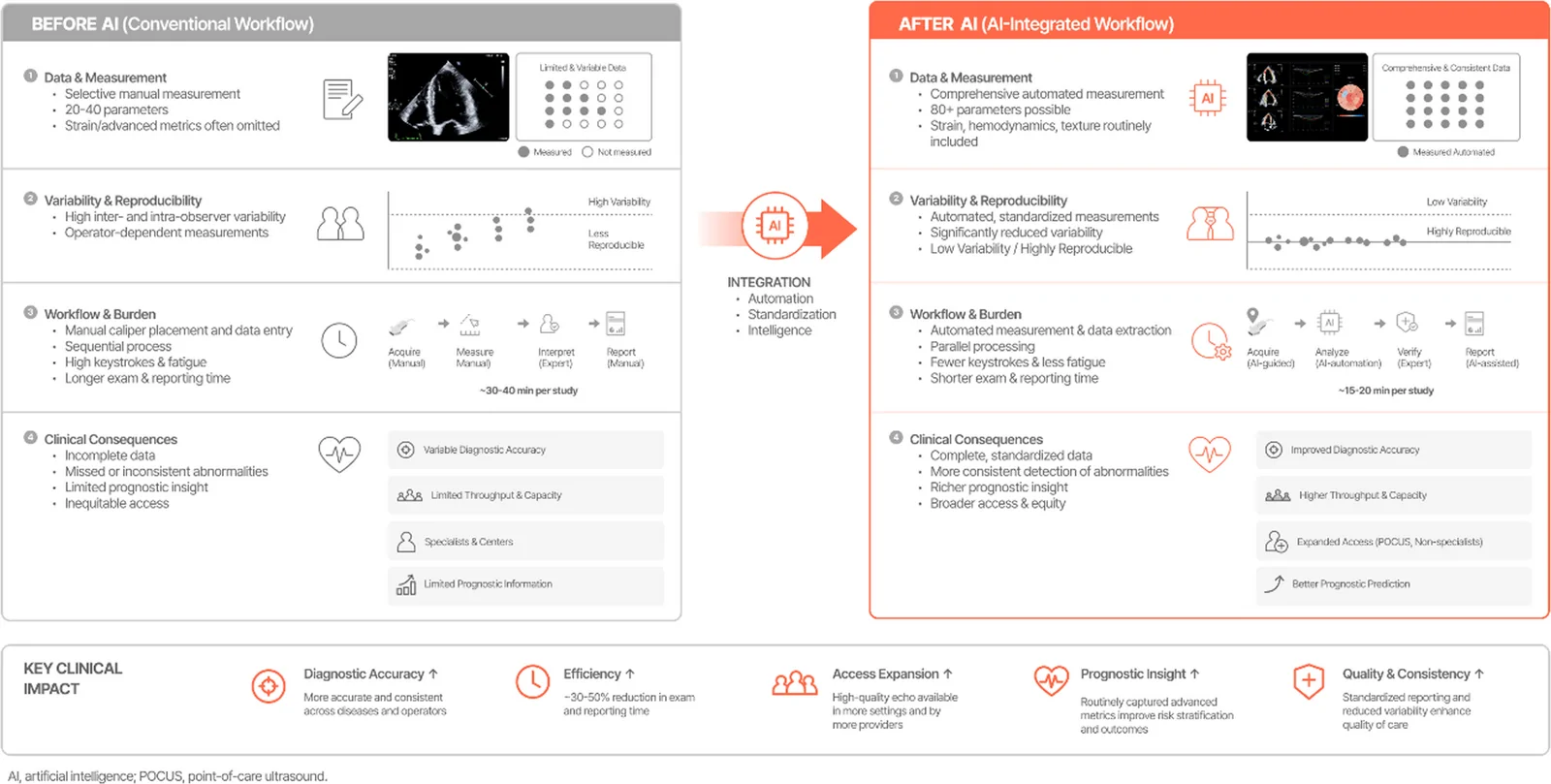
Clinical impact of artificial intelligence (AI) integration in echocardiography: from measurement-limited to data-rich and consistent care. Conventional echocardiography is characterized by selective, manual measurement, leading to incomplete data capture, inter-operator variability, and time-intensive workflows. AI integration enables comprehensive and standardized parameter extraction, reduces variability, and improves workflow efficiency. These changes translate into improved diagnostic consistency, expanded access to echocardiography, and potential prognostic enrichment from more complete parameter capture

Automated LVEF measurement, while clinically important, represents only one component of a comprehensive echocardiographic examination. The most consequential—and most recent—developments in this field lie in AI's extension to parameters requiring greater interpretive complexity: myocardial deformation, multitask diagnostic classification, and disease-specific reasoning, as summarized in Fig. 3. Largely reported between 2025 and 2026, these developments mark the point at which AI moves from automating what a sonographer already measures toward approximating how a cardiologist reasons.

### Automated strain analysis: guideline-endorsed parameters

Major guideline frameworks now include strain in routine echocardiographic assessment. The 2025 American Society of Echocardiography (ASE) update on LV diastolic function includes left atrial reservoir strain as an adjudicating parameter in inconclusive diastolic assessments [8], extending the 2016 ASE and European Association of Cardiovascular Imaging (EACVI) framework [9] in line with the 2022 EACVI expert consensus document [10] and 2024 British Society of Echocardiography recommendations [11]. The 2022 cardio-oncology guidelines by the European Society of Cardiology define cancer therapy-related cardiac dysfunction using both LVEF and global longitudinal strain, establishing strain as a standard surveillance endpoint in patients receiving potentially cardiotoxic therapies [12]. Despite this guideline endorsement, strain analysis has historically been limited by the need for dedicated speckle-tracking software, additional postprocessing time, and vendor-specific measurement conventions—barriers that have restricted its routine use outside specialized centers.

A recurring insight across these workflow and measurement studies is that the practical value of AI may lie less in superior measurement of any single parameter than in the routine completeness of its output—automatically capturing parameters, such as strain, that are clinically important but inconsistently obtained in conventional practice.

A retrospective single-center study of 731 hospitalized patients found that AI-based and human expert echocardiographic analysis showed comparable mortality prediction using standard parameters (area under the curve [AUC], 0.67 vs. 0.66; *P* = 0.86) [13]. However, when strain—automatically included in AI-analyzed studies but not routinely obtained in human analysis—was added to the AI model, prognostic performance improved significantly (AUC, 0.73 vs. 0.66; *P* = 0.048), reinforcing the pattern described above—a principle that recurs across the more diagnostically complex applications discussed below. However, 18% of studies were excluded—approximately 8% due to insufficient image quality for complete AI analysis and the remainder for unrelated data-completeness or technical reasons—a limitation particularly relevant in populations where strain is most clinically important, such as cardio-oncology patients with nondiagnostic acoustic windows.

### Multitask AI: comprehensive echocardiographic interpretation

Holste et al. [14] presented a multitask deep learning model trained on 1.2 million echocardiographic videos from 32,265 studies at Yale New Haven Health System, designed to perform 18 diagnostic classification tasks and estimate 21 parameters simultaneously from a standard TTE. Across internal and external validation cohorts, the model achieved a median AUC of 0.91 for classification tasks, with strong performance for LV systolic dysfunction (moderate or worse left ventricular systolic dysfunction: AUC, 0.98 internal and 0.99 external; severe aortic stenosis: AUC, 0.98 internal and 1.00 external), and LVEF estimation (mean absolute error, 4.2% internal; 4.5% external). Performance was maintained in emergency department point-of-care ultrasound (POCUS) acquisitions (median AUC, 0.85 across 14 tasks), suggesting applicability beyond the controlled laboratory setting.

Generating a comprehensive parameter set in a single step reduces sequential measurement burden but raises interpretability concerns. When a deep learning model predicts severe aortic stenosis or LV dysfunction directly from raw video pixels, without providing traditional Doppler envelopes or traceable contours, clinicians have no mechanism to visually verify the output—limiting clinical trust and adoption. This tension between predictive performance and inspectable, verifiable output recurs throughout the diagnostic applications below, and is addressed directly in the discussion of explainability and verification that follows.

### Beyond LVEF: the shift towards explainable hemodynamics and pathology

AI applications have successfully extended to structural conditions, including the diagnostic grading and prognostic prediction of valvular heart disease and phenotyping or classification of etiology [15–19]. However, as with the multitask models discussed above, early deep learning models in this space largely relied on the same unverifiable, pixel-based predictions, limiting clinicians' ability to verify the output.

Recent models have moved beyond purely intuitive image classification toward integration of objective, multiparametric clinical evidence. Park et al. [20] developed an AI framework for aortic stenosis severity grading that incorporates recognition and quantitative analysis of continuous-wave Doppler velocity patterns alongside structural imaging, synthesizing anatomical and hemodynamic data. This approach demonstrated utility for both diagnostic grading and longitudinal prognostic prediction [16]. Models that incorporate measurable, familiar data points such as Doppler patterns may enhance both diagnostic precision and clinical trust by providing verifiable, interpretable outputs.

For cardiomyopathy and myocardial infiltration, early AI approaches demonstrated that deep learning models trained on large multicenter video datasets could accurately classify disease across diverse ethnic and institutional contexts [21]. However, these models operate as opaque classifiers, replicating a diagnostic label without revealing which image features drove the decision.

A more recent direction asks AI to formalize and quantify the subtle visual features that experienced cardiologists recognize intuitively but have historically been difficult to measure. The "granular sparkling" texture of the amyloid-infiltrated myocardium is a representative example. Myocardial texture analysis enables automated extraction of pixel-level statistical features, including gray-level co-occurrence matrices and neighborhood gray-tone difference matrices, that formally capture altered myocardial echogenicity, with Datar et al. [22] demonstrating proof-of-concept for detection. A complementary approach applies direct pixel intensity quantification to differentiate transthyretin amyloid cardiomyopathy from amyloid light-chain amyloidosis and other phenocopies that are visually indistinguishable to most clinicians [23]. Moon et al. [24] extended both principles across four echocardiographic views, integrating myocardial texture and geographic features into a machine-learning framework with Shapley Additive Explanation–based interpretability.

For pericardial disease, AI addresses the differentiation of constrictive pericarditis from restrictive cardiomyopathy—one of the challenging distinctions in echocardiography. The two conditions share overlapping hemodynamic and morphological features yet carry different prognoses—constrictive pericarditis being surgically curable if identified in time. Chao et al. [25] demonstrated that a deep learning model applied to a single apical four-chamber view could differentiate constrictive pericarditis from restrictive cardiomyopathy with robust external validation performance, providing a platform for earlier referral for pericardiectomy.

Beyond this binary distinction, Jeong et al. [26] developed an AI framework to evaluate the full clinical spectrum of pericardial disease. Rather than treating pericardial assessment as a single classification task, the model incorporates sequential reasoning across two stages: the first stage evaluates morphological features—effusion grading and pericardial thickening or adhesion—from multiple B-mode views, while the second stage integrates Doppler and inferior vena cava measurements to assess hemodynamic significance. This approach reflects the experienced clinician’s reasoning, integrating morphological and hemodynamic data to reach a comprehensive assessment.

Across valvular disease, cardiomyopathy, and pericardial disease, these efforts share a common trajectory: AI is moving from replicating a diagnostic label learned from raw video data toward formalizing the perceptual and hemodynamic reasoning that expert clinicians already apply intuitively—quantifying myocardial texture, anchoring predictions to measurable Doppler patterns, and structuring multistage reasoning across morphology and hemodynamics. If this trajectory continues, the next generation of AI models in echocardiography may be defined less by diagnostic accuracy alone than by the degree to which their outputs remain clinically inspectable and verifiable—the theme addressed directly in the section that follows.

### Explainability, verification, and the changing roles of sonographers and cardiologists

This reconfiguration is reflected in a new division of labor: sonographers focus on high-quality image acquisition while AI performs measurement in the background, and cardiologists supervise and synthesize an expanded parameter set. The measurement task shifts from active performance to active verification, modifying the cognitive demands of both roles without eliminating either.

#### Explainability versus clinical verifiability

Post hoc explainability, such as saliency maps highlighting the image regions that most influenced a model's output, is not equivalent to a clinically verifiable output. In echocardiography, genuine verification depends on inspectable elements: view classification, endocardial contours, Doppler envelopes, image quality and confidence scores, and explicit failure alerts. Models that predict directly from raw video pixels, as discussed above, can be explained after the fact but cannot always be verified against these familiar clinical reference points.

As AI systems advance toward drafting comprehensive and structured reports via large language models (LLMs) [27, 28], the risk of automation bias becomes relevant—the tendency of clinicians to accept machine-generated outputs without adequate scrutiny, particularly in high-volume environments. When an AI prepopulates a complex echocardiography report, the cardiologist's role risks shifting from active interpretation to passive acceptance.

Mitigating this risk requires intentional design: user interfaces that demand active verification, explainable AI that links measurements back to the original acoustic windows, and diagnostic confidence scores where appropriate.

For the present, LLM-based reporting is best limited to structured drafting from verified measurements rather than autonomous free-text interpretation. Legal and ethical liability remains with the interpreting cardiologist, and governance frameworks for AI-assisted reporting should empower physicians to exercise clinical judgment efficiently rather than bypass it.

## Democratization of echocardiography: nonspecialist use, POCUS, and expanded access

Beyond changes in workflow and diagnostic capability, AI is also reshaping who can perform and access echocardiographic assessment: extending it from specialist laboratories to nonspecialist clinicians, point-of-care settings, and resource-limited environments.

### AI-guided acquisition and educational transformation

The utility of AI extends beyond postprocessing and interpretation to the direct point of care through real-time image acquisition guidance. Historically, the extensive training required for echocardiography has limited its comprehensive use by nonspecialists. Narang et al. [29] demonstrated that nurses without prior ultrasonography experience could acquire diagnostic-quality echocardiographic images using an AI-guided system following brief training, suggesting potential applicability for other nonspecialist users including medical students and internal medicine physicians.

### Extension of echocardiography beyond the specialist laboratory

#### Impact on clinical decision-making by nonspecialist physicians

A prospective trial evaluated AI-assisted bedside echocardiography by nonspecialist physicians in hospitalized patients [30]. Treatment plan changes were more frequent in the AI-assisted group, suggesting that AI interpretation can influence clinical management. Whether these changes were appropriate and associated with improved outcomes requires further study.

#### Handheld AI echocardiography in heart failure pathways

A prospective multicenter study evaluated fully automated AI analysis of handheld echocardiography in 587 patients with suspected heart failure across five outpatient clinics, without exclusions for poor image quality [31]. The system identified LVEF ≤ 40% with an AUC of 0.96 (sensitivity, 0.61; specificity, 0.95; negative predictive value, 0.97), and LVEF estimates were interchangeable with those from expert-performed echocardiography. The high negative predictive value supports handheld AI echocardiography as a triage or rule-out tool in heart failure diagnostic pathways—rather than a substitute for diagnostic transthoracic echocardiography—with positive or indeterminate findings prompting escalation to formal TTE. The sensitivity of 0.61 indicates that a meaningful proportion of cases with reduced LVEF may not be detected, particularly in patients with suboptimal image quality.

#### AI-assisted LVEF monitoring by oncology staff

One study evaluated whether oncology staff—a senior oncologist, junior oncologist, and nurse—could accurately measure LVEF using an AI-enabled handheld device in 115 patients following standardized training [32]. All three operator types achieved acceptable agreement with cardiologist-performed echocardiography. This model could facilitate serial LVEF monitoring within the oncology clinic setting, though the single-center design and structured training conditions limit current generalizability.

### Economic considerations and access to echocardiography

An economic evaluation modeled the cost-effectiveness of AI-based LVEF task-shifting in Singapore, demonstrating economic benefit through reduced specialist time per examination [33]. Kusunose [34] described AI-enhanced tele-echocardiography as a practical model for delivering cardiac imaging in geographically isolated communities with limited physician resources. Together, these findings suggest that AI may support wider availability of echocardiography, though evidence from lower-resource settings remains limited.

## Current limitations and conditions for responsible implementation

The body of evidence reviewed above supports the potential of AI to improve echocardiographic workflow efficiency, expand diagnostic scope, and extend access. However, several limitations must be acknowledged before broad clinical generalization.

### Characteristics of the current evidence base

Most workflow studies involve small numbers of sonographers or cases at single academic centers. The AI-Echo RCT study, while randomized, enrolled four sonographers performing screening examinations at one institution [3]. The ergonomic study by Hollitt et al. [1] and the LLM study by Syryca et al. [27] are similarly limited in scale. These studies provide directional evidence, but generalization to different clinical contexts, such as varied examination types, diverse sonographer experience levels, and lower-resource settings, requires multicenter studies with larger and more representative samples.

#### Parameter-specific variability in AI performance

AI performance differs across echocardiographic parameters. LVEF from standard apical views is the most comprehensively validated domain. Diastolic function grading, which requires integration of multiple Doppler indices with interpretive nuance, and right ventricular parameters present greater challenges. Valvular disease severity grading remains variably validated across specific lesions. Clinicians deploying AI tools should understand which parameters are reliably automated on a given platform and which require human verification.

#### Intervendor variability in AI measurements

Szasz et al. [35] demonstrated that different AI platforms do not produce consistent measurements for the same echocardiographic studies. Switching platforms without recalibration, or combining AI measurements from different vendors in serial monitoring, risks introducing systematic errors with clinical consequences.

#### Dependence of AI performance on image quality

Most validated systems perform best in studies of adequate image quality. The blinded LVEF trial excluded approximately 7% of screened studies for poor image quality [5]; real-world exclusion rates in unselected populations may be higher. AI performance on studies with suboptimal acoustic windows, motion artifact, or unusual anatomy is less well characterized than on curated high-quality datasets.

#### Regulatory and liability considerations

AI systems generating diagnostic measurements are regulated as Software as a Medical Device in most jurisdictions. Clearance for a specific validated use case does not extend to all deployment contexts. The question of liability—who bears responsibility when an AI-generated measurement contributes to patient harm—remains unresolved in most legal frameworks. Current practice assigns ultimate responsibility to the interpreting cardiologist, requiring genuine oversight rather than passive acceptance of AI outputs [36].

#### Implications for professional education and training

Effective use of AI-integrated echocardiography requires that sonographers and cardiologists understand the performance characteristics and failure modes of the systems they employ. AI literacy is not yet systematically addressed in training curricula for either profession. As AI becomes more deeply integrated into echocardiographic practice, this gap will need to be addressed in formal training programs.

To translate these limitations into practical implementation steps, echocardiography laboratories adopting AI should establish a structured governance process before routine deployment. A practical checklist for responsible laboratory adoption is summarized in Table 3.

**Table 3:** Practical checklist for responsible AI adoption in echocardiography laboratories

Domain	Checklist item
Local validation	Validate the AI system on representative local studies before deployment, including the laboratory’s scanners, acquisition protocols, patient population, sonographer experience levels, and image quality spectrum
Human-in-the-loop verification	Define which AI outputs can be used routinely, and which require closer review. Clinicians should actively verify key measurements, contours, Doppler envelopes, confidence scores, and report drafts before finalization
Quality assurance and drift monitoring	Establish a process to audit discordant cases, failed analyses, low-confidence outputs, and cases requiring manual correction. Reassess performance after software updates, hardware changes, transducer replacement, or major workflow changes
Platform consistency	Interpret serial measurements with awareness of intervendor and interplatform variability, especially for LVEF, chamber volumes, strain, Doppler-derived indices, and valvular parameters
Appropriate clinical scope	Use AI-guided handheld or nonspecialist echocardiography as triage or rule-out support rather than as a substitute for comprehensive diagnostic transthoracic echocardiography. Escalate to standard echocardiography when image quality is inadequate, AI confidence is low, results are abnormal or borderline, or findings are discordant with the clinical presentation
Reporting and accountability	Limit LLM-based reporting to structured drafting from verified measurements, predefined report elements, and clinician-approved templates. The interpreting physician should remain responsible for the final report, and autonomous free-text interpretation by LLMs should be avoided

AI, artificial intelligence; LVEF, left ventricular ejection fraction; LLM, large language model

## Conclusions

The evidence reviewed here supports a field in transition. Randomized trials confirm that AI-based measurement reduces operator-dependent variability and expands diagnostic parameter coverage without lengthening examinations. Multitask systems demonstrate comprehensive interpretive capability across diverse clinical scenarios, and AI-enabled devices extend echocardiographic assessment to point-of-care and nonspecialist settings.

As introduced at the outset, the more consequential development is qualitative rather than quantitative. AI in echocardiography is evolving from unexplained pattern recognition toward architectures that formalize and externalize expert clinical reasoning—quantifying myocardial texture, anchoring predictions to Doppler hemodynamics, and encoding the structural-then-functional logic that experienced cardiologists apply intuitively. This shift matters because it addresses the core barrier to clinical trust: explainability, and, as distinguished above, genuine clinical verifiability.

Realizing the potential of AI in echocardiography will require multicenter prospective validation, cross-platform standardization, and the formal integration of AI literacy into cardiovascular training. AI technology is maturing; the governance frameworks and educational infrastructure must now keep pace.

## Data Availability

No datasets were generated or analysed during the current study.

## Abbreviations

AI: Artificial intelligence
ASE: American Society of Echocardiography
AUC: Area under the curve
EACVI: European Association of Cardiovascular Imaging
LLM: Large language models
LV: Left ventricle
LVEF: Left ventricular ejection fraction
POCUS: Point-of-care ultrasound
TTE: Transthoracic echocardiogram
